# Clinical effectiveness of drop-in mental health services at paediatric hospitals: A non-randomised multi-site study for children and young people and their families – study protocol

**DOI:** 10.1371/journal.pone.0302878

**Published:** 2024-05-09

**Authors:** Anna Roach, Sophie Bennett, Isobel Heyman, Anna Coughtrey, Isabella Stokes, Xhorxhina Ndoci, Sonia Balakrishnan, Nicki Astle, Jessie Drinkwater, Rebecca Evans, Una Frederick, Michael Groszmann, Steve Jones, Katie McDonnell, Amanda Mobley, Abbie Murray, Helena O’Sullivan, Sarah Ormrod, Theodore Prendegast, Usha Rajalingam, Emily Webster, Rebecca Webster, Gareth Vinton, Roz Shafran

**Affiliations:** 1 UCL Great Ormond Street Institute of Child Health, London, United Kingdom; 2 Great Ormond Street Children’s Hospital, London, United Kingdom; 3 Department of Psychology, Institute of Psychiatry Psychology & Neuroscience, King’s College London, De Crespigny Park, London, United Kingdom; 4 Cambridge Children’s Hospital Project Team and Paediatric Psychological Medicine Service, Addenbrooke’s Hospital, Cambridge, United Kingdom; 5 University College Hospitals, London, United Kingdom; 6 Hinchingbrooke Hospital, Hinchingbrooke Park, Huntingdon, Cambridgeshire, United Kingdom; 7 Sheffield Children’s Hospital, Sheffield, United Kingdom; 8 Peterborough City Hospital, Edith Cavell Campus, Bretton Gate, Peterborough, United Kingdom; 9 Leeds Teaching Hospital Trust, Paediatric Psychology, E Floor Martin Wing, Leeds General Infirmary, Leeds, United Kingdom; 10 Cambridge and Peterborough Foundation Trust, Child Development Centre, City Care Centre, Peterborough, United Kingdom; Jawaharlal Institute of Postgraduate Medical Education and Research, INDIA

## Abstract

**Background:**

Despite the high prevalence of mental health difficulties in children and young people with long-term health conditions (LTCs), these difficulties and experiences are often overlooked and untreated. Previous research demonstrated the effectiveness of psychological support provided via a drop-in mental health centre located in a paediatric hospital. The aim of this prospective non-randomised single-arm multi-centre interventional study is to determine the clinical effectiveness of drop-in mental health services when implemented at paediatric hospitals in England.

**Methods:**

It is hypothesised that families who receive psychological interventions through the drop-in services will show improved emotional and behavioural symptoms. Outcomes will be measured at baseline and at 6-month follow-up. The primary outcome is the difference in the total difficulties score on the Strengths and Difficulties Questionnaire (SDQ) reported by parent or child at 6 months. Secondary outcomes include self and parent reported Paediatric Quality of Life Inventory (PedsQL), self-reported depression (PHQ-9) and anxiety measures (GAD-7) and family satisfaction (CSQ-8).

**Discussion:**

This trial aims to determine the clinical effectiveness of providing psychological support in the context of LTCs through drop-in mental health services at paediatric hospitals in England. These findings will contribute to policies and practice addressing mental health needs in children and young people with other long-term health conditions.

**Trial registration:**

ISRCTN15063954, Registered on 9 December 2022.

## Background and rationale (6a)

In the UK, approximately 23% of children and young people (CYP) are living with a long-term health condition (LTC), defined as any diagnosed health condition lasting for a minimum of three months, for which a cure is unlikely and results in limitations in ordinary activities and increased use of health services [1,2]. Living with a LTC can have adverse impacts on CYP’s daily functioning, such as difficulties managing emotional, educational, behavioural and social needs, and can have adverse impacts on their quality of life [3,4].

CYP with LTCs are at a greater risk of developing mental health problems such as depression, anxiety and disruptive behaviour disorder when compared to their physically healthy peers [1,5–7]. Despite regular contact with healthcare providers for physical health symptoms, CYP’s mental health concerns are often overshadowed and overlooked If untreated, mental health conditions can be associated with poor adherence to medication [8], negative physical health outcomes [9] and increased hospital stays [10].

The impact of living with a LTC also extends to CYP’s family members, with higher levels of stress and mental health symptoms reported in parents and elevated emotional issues in siblings [11,12].

Evidence-based treatments for mental health problems have been shown to be effective in CYP with LTCs [13,14]. Recent systematic reviews found that NICE recommended treatment, cognitive behavioural therapy (CBT), demonstrated positive outcomes for psychiatric symptoms in children with mental health difficulties in the context of LTCs [1,15]. That said, there are several barriers that limit access to evidence-based mental health care for CYP with LTCs. These include a lack of trained therapists, work demands, time, transport and costs alongside a lack of understanding that CYP with LTCs respond to existing evidence-based treatments [16–18]. There are also structural barriers in terms of service support for example, who is permitted as a referrer, and increasingly stringent referral criteria for services with referral outcomes for CYP with LTCs often unknown [19]. When care is offered, mental and physical health services are typically separate and not well integrated One suggested strategy to improve timely and flexible access to mental health interventions for CYP with LTCs is the integration of psychological care into paediatric hospitals [20].

Another way to overcome barriers to accessing mental health support is through the introduction of brief and low intensity psychological interventions. Low intensity CBT has been defined as utilising guided self-help materials, with six hours or less of contact time, with each contact typically 30 minutes or less, where input can be provided by trained practitioners or supporters [21]. These specialised interventions address potential time constraints that patients or hospital services may report, provide a route to administer evidence-based interventions in a more accessible manner that can be helpful for patients that may not meet stringent CAMHS criteria for high intensity interventions, but would still benefit from psychological support. Additionally, given that low intensity interventions do not usually require the same length of training as that required for high intensity intervention, more staff can be trained specifically to provide these services within a solid, evidence-based framework of structured supervision, over a shortened period of time.

A model of delivering low intensity psychological support to families with LTCs is to use a ‘drop-in centre’ located within a paediatric hospital, offering support at point of need with limited waiting time. Such support includes signposting to existing provision, referral to appropriate services, neurodevelopmental assessments, as well as the provision of low intensity CBT. Previous research indicated that this can be an effective way of providing mental health support for families in the context of LTCs, reducing emotional and behavioural symptoms and improving reported quality of life for CYP and their families [14,22]. Despite encouraging evidence, it is important to understand if (and how) this model can be implemented in different paediatric hospitals and services in England. Furthermore, research often fails to quickly translate into routine clinical practice. The implementation of a new intervention can take up to 17 years to be incorporated into practice and it is essential to consider factors that can facilitate this process [23,24]. The drop-in services may vary operationally from the original research project, and differ at each new site although key principles will be retained i.e., self-referral, integration with existing services, use of evidence-based interventions, any direct provision being low intensity. The COVID-19 pandemic increased the use of telehealth and digital appointments as hospital footfall dropped [25], therefore the drop-in “centres” will instead operate as drop-in “services” offering face-to-face, digital and telephone appointments depending on patient preference. To roll-out these services researchers will closely work with new hospitals to best understand how this can be delivered within their existing teams, pathways and capacities.

### Objectives (7)

The overarching objective of this research is to implement drop-in services for CYP and families with mental health needs in the context of LTCs at various different paediatric hospitals in England. This single-arm trial aims to evaluate the clinical effectiveness of the drop-in services in providing access to mental health support. The drop-in services will be integrated with existing mental health provision and serve as a single point of access delivering evidence-based low intensity psychological interventions or signposting and onward referral where clinically appropriate.

### Trial design (8)

This present study is a prospective non-randomised single-arm multi-centre interventional trial investigating the clinical effectiveness of drop-in mental health services at paediatric hospitals. Patients aged up to 25 years old with a LTC, who have been receiving care for their LTC for 6 months or more, and their parent/carer and siblings can take part in the trial. The primary outcome is the difference in the total difficulties score on the Strengths and Difficulties Questionnaire (SDQ) reported by parent or child at 6 months.

## Methodology: Participants, interventions and outcomes

The trial was registered at ISRCTN (ISRCTN15063954) on 9th December 2022. The trial was registered two weeks after enrolment began as approval for participating hospitals to begin recruitment was granted sooner than expected and the research team wished to adapt to the hospital’s respective timelines. The authors confirm that all ongoing and related trials for this intervention are registered.

### Study setting (9)

Recruitment will take place at any of the participating paediatric hospitals in England. For a full list of the participating sites, please visit https://www.isrctn.com/ISRCTN15063954.

### Eligibility criteria (10)

Participants will be eligible to participate in this study if they meet the below inclusion criteria.

### Participant inclusion criteria

1. Patients aged between 0–25 years old, or a parent/carer or sibling of the patient.
2. Receiving care for a LTC at one of the participating hospitals.
3. Receiving care for their LTC 6 months or more.

#### Exclusion criteria

Participants included in the study analysis cannot be receiving another form of concurrent psychological care from the hospital. Participants are eligible if on a waiting list for psychology support.

### Who will take informed consent? (26a)

Consent will be completed online or taken over the phone. The order of referral procedures and the consent process is as follows:

1. Participants will be referred to the drop-in service through different routes depending on their hospital. For instance, self-referrals to the service can be made by using the QR code or email address which are located on posters in the hospitals. CYP can also be referred by their clinician who, upon receiving their consent to contact, can share contact details with the study team.
2. The study team will get in contact with the participant (or parent/carer when appropriate) to provide more information and details of the consent process.

#### Confidentiality

Identifiable information pertaining to the participant is essential for participant registration. Anonymity will be held unless any information disclosed about the participant indicates that they are in a situation posing risk to themselves or others. If risk is identified, the research team will inform the participant’s GP and hospital site.

### Intervention description

Once consented and in contact with the research team, the participant will provide demographic information and complete initial baseline measures.

1. Parent-reported Strengths and Difficulties Questionnaire, and/or self-reported for CYP aged 11+ (SDQ) [26]
2. Parent-reported Paediatric Quality of Life Questionnaire, and/or self-reported for CYP aged 8+ (PedsQL) [27]
3. Self-reported Patient Health Questionnaire, for parents and CYP aged 12+ (PHQ-9) [28]
4. Self-reported Generalized Anxiety Disorder Assessment, for parents and CYP aged 12+ (GAD7) [29]

**Fig 1:**
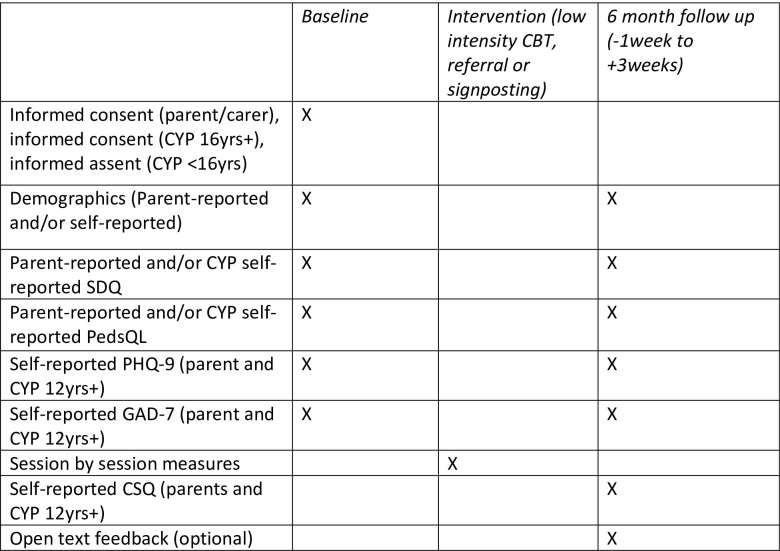
SPIRIT schedule of participant timeline.

After baseline measures and consent have been completed, the participant’s GP and hospital site will be informed and consent forms and baseline measures, including any identified risk, will be shared. An assessment appointment with the participant will be set up by the low intensity trained practitioner from the hospital site. This assessment is used to help decide, in collaboration with the family, which intervention allocation is most suitable for the participant. The assessment will be discussed in supervision provided by a clinical psychologist, or a senior low intensity practitioner under the supervision of a clinical psychologist. After this discussion patients will either be assigned to receive an evidence-based low intensity CBT intervention (selected from a set of empirically validated options) or will be signposted or referred to other services in their hospital or local area. If the participant is suitable for low intensity CBT, they will receive weekly sessions face-to-face, over the telephone or over video call depending on participant preference, lasting around 30–45 minutes for 6–8 weeks with a trained practitioner. If a low intensity intervention is deemed to not be the most appropriate allocation for the participant’s needs, they will be referred to a different service, such as CAMHS or hospital psychology services, or they will be signposted to external services, for example a charity or non-profit organisation. At six months post baseline assessment an independent researcher will contact participants with follow up questionnaires to be completed over the phone or online. CYP and/or their parents (depending on age) will complete the SDQ, PedsQL, PHQ-9, GAD-7 and a modified Centre Satisfaction Questionnaire (CSQ) [30]. This process is outlined in Fig 1.

### Patient Public Involvement (PPI)

The Young People’s Advisory Group (YPAG) at Great Ormond Street Hospital and the Cambridge Parents’ Advisory Group have, and will continue to be, consulted throughout this project. PPI input has been invaluable, providing advice on patient facing materials, online questionnaires and dissemination. Parent and child PPI meetings were held separately to provide a comfortable environment for parents and children to share their opinions and to appreciate the potential differences in questions, concerns, and interests that each group may have.

### Criteria for discontinuing or modifying allocated interventions (11b)

When participants consent to the trial, they will be informed that they are consenting to the trial intervention, trial follow-up and data collection. If at any point the following issues occur with a participant, the intervention will be terminated for that participant:

- Participant’s withdrawal of consent from the trial intervention
- Participant’s illness inhibits continual engagement with the intervention, in a judgement made from the discretion of the clinician

As participation in the trial is entirely voluntary, the participant may choose to discontinue trial treatment at any time without penalty or loss of benefits to which they would otherwise be entitled.

### Training and supervision of practitioners

It has been found that a lack of adequate training resources is a possible factor contributing to implementation challenges of low intensity psychological interventions for children and young people [31]. To address this issue, the current project has delivered training on low intensity psychological interventions for CYP in the context of LTCs for clinicians from participating hospitals [32]. Hybrid training was provided by a clinical psychologist and PhD student over two days to a wide range of clinicians, including non-mental health specialists. The training covered techniques such as information gathering and guided self-help, how to equip patients with LTCs who are experiencing mental health difficulties (and their families) and shared evidence-based low-intensity CBT strategies to improve low mood, anxiety and behavioural difficulties. The training was recorded using Microsoft Teams, making it easily accessible for practitioners to refer back to and for future hospital sites to acquire the training in a timely manner.

Across the different sites, different practitioners will provide low intensity CBT to participants. Experience will vary with some practitioners already qualified low intensity practitioners (and therefore have completed in depth training and have post qualification experience), and others who are new to CBT. Supervision and consultation will be adapted according to experience.

### Strategies to improve adherence to interventions (11c)

All practitioners delivering low intensity interventions will have access to regular weekly consultation. This will be provided by a clinical psychologist or a senior low intensity practitioner under the supervision of a clinical psychologist. Clinical supervision will be provided by their own hospital site and the research team will be available for consultation on using the evidence based low intensity manuals and/or any study processes.

Sites will be provided with evidence-based guided self-help manuals to use in their sessions. Sites may have different treatments options available, and sites will also have access to a shared document outlining their evidence base and clinical recommendations.

### Relevant concomitant care permitted or prohibited during the trial (11d)

Participants who are actively receiving any other psychological interventions for behavioural and emotional difficulties will not be permitted to participate in the trial.

### Provisions for post-trial care (30)

There are no plans for ancillary or post-trial care. Any significant risk issues will be communicated to the families’ GP and any other clinician involved in their care. Referrals or requests for referrals to other services will be made as appropriate.

### Outcomes (12)

#### Primary outcome

The primary outcome is the difference in the total difficulties score on the Strengths and Difficulties Questionnaire (SDQ) reported by parent or child at 6 months The appropriate form was used depending on the age of the child. (SDQ self-report 11–17 years, SDQ self-report 18 years and older, SDQ parent report 2–4 years, SDQ parent report 4–17 years, SDQ informant report 18 years and older) [26,33]. This questionnaire assesses five subscales (prosocial, hyperactivity, peer problems, emotional problems, conduct problems) in addition to the total difficulties score. The SDQ is a commonly used questionnaire for children and young people with robust psychometric properties [34].

### Secondary outcomes

- The Paediatric Quality of Life Inventory (PedsQL) [27] (Parent-rated versions are available for children aged 2–4, 5–7, 8–12 and 13–18 years and self-report versions are for young people aged 5–7, 8–12, 13–17, 18–25 years [35,36])
- Patient Health Questionnaire (PHQ-9) [28]
- The Generalized Anxiety Disorder (GAD-7) [29]
- SDQ Impact [26]
- Client Satisfaction Questionnaire (CSQ-8) [30]

### Sample size (14)

The sample size is 109+11 children and young people with a LTC and/or their families. This was calculated using the SDQ population averages for young people and previously published research on similar interventions and their effect on SDQ score [14,37]. Previous published research has estimated the effect size for usual care in UK CAMHS to be 0.16–0.2 [38]. The total sample size of 120 could detect a small to moderate effect size, at 5% significance level with 80% power, with a loss to follow-up rate of 10%. It is estimated that 10 participants will be recruited each month over a period of 12 months.

### Recruitment (15)

Participants will be able to join the study through various routes. Participants can self-refer into the study using the QR code or email address on the poster and newsletters advertising the drop-in service in participating hospitals. Alternatively, participants may be referred to the service through their clinician and after they have provided consent to contact, patient details will be given to the research team. Eligibility criteria will be checked and then participant information sheets can be shared. If interested, participants can then consent to take part and complete baseline measures. After these are collected, an assessment will be organised with a low intensity practitioner, discussed in supervision and appropriate intervention will be allocated.

The aim is for all participants to be offered an assessment within two weeks after consenting to ensure the service is providing “drop-in” support and timely access to treatment.

### Assignment of interventions

**Fig 2:**
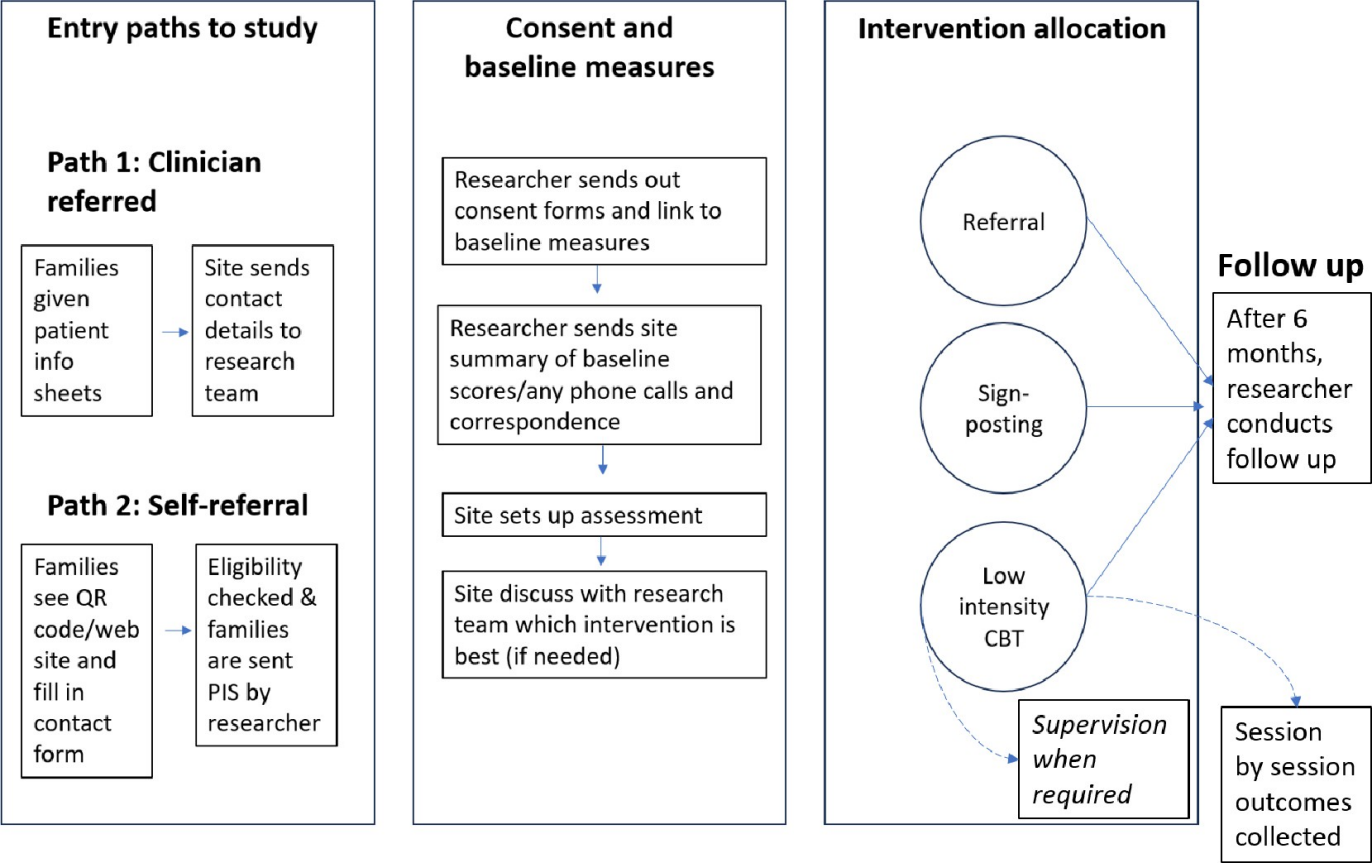
Participant pathways from recruitment to follow up.

There are three types of intervention allocations: low intensity CBT, onward referral or signposting (Fig 2). After the practitioner has discussed the assessment with their supervisor, CYP who display mild to moderate emotional and/or behavioural difficulties will be offered low intensity CBT. For CYP where low intensity CBT may not be most clinically appropriate (e.g. if their mental health concern is surrounding adjustment to new condition or a bereavement, or if they have previously had low intensity CBT for the same problem), individuals may be referred to other therapy modalities (such as narrative therapy, family therapy or counselling), or signposted towards other services (e.g., a local support group or charity). Parents or siblings over 25 years old who take part in the study may be offered a one-off CBT session (as practitioners are trained to deliver low intensity CBT sessions for CYP mental health difficulties rather than adult mental health difficulties) or referred or signposted for further support. Regardless of treatment allocation, all participants who consented and completed baseline measures will be followed up after 6 months.

## Data collection and management

### Plans for assessment and collection of outcomes (18a)

Participants that provide consent to take part in the study will be assigned a unique participant identification number. Data collected at baseline and 6-month follow up will be collected over the phone, face-to-face or online using REDcap depending on participants’ preferences. REDCap [39,40] is a secure electronic data capture and transfer software developed by Vanderbilt University. The software is hosted on virtual servers provided by AIMES (an ISO27001 certified cloud hosting provider) in its own data centre in Liverpool, UK. The software is licenced for use by Vanderbilt University to REDCap consortium members, of which Great Ormond Street Hospital is one. The study data will be stored on secured virtual Linux servers (specifically GOSH-REDCap-DB01, GOSH_REDCap-WB01) with an appropriate level of encryption based in England, UK. All data will be handled in accordance with the General Data Protection Regulation and UK Data Protection Act 2018.

### Data management (19)

Each participant will be allocated a unique study number at study entry. They will be identified by this number on all study related documentation throughout the course of the intervention and data analysis process. Any data transferred will be done according to the NHS Code of Practice on Confidentiality. Access to the database is controlled by password-protected accounts, and an individual’s access to the database is restricted by their role.

### Plans to promote participant retention and complete follow-up (18b)

The importance of attending intervention sessions and completing follow-up measures will be explained to all participants at the start of the study. Participants who discontinue treatment should remain in the study for the purpose of follow-up and data analysis. They will be asked whether they continue to consent to being contacted for follow-up measures. Data will be analysed on an intention-to-treat basis. Therefore, withdrawal from the study will not result in exclusion of the data for that participant from analysis.

## Statistical methods

### Statistical methods for primary and secondary outcomes (20a)

Patient characteristics at baseline will be summarised using mean and standard deviation (SD) for continuous variables which are approximately normally distributed, or median and interquartile range (IQR) for variables which are not normally distributed, or by frequencies and percentages for categorical variables. All statistical hypothesis tests will use a two-sided p-value of 0.05 unless otherwise specified. All confidence intervals (CIs) presented will be 95% and two-sided. All statistical analysis will be performed using SPSS statistics analysis software (V.25, IBM) or an alternative package.

### Primary outcome

The primary outcome is the difference in the total difficulties score on the SDQ reported by parent or child at 6 months. The total difficulties score of the SDQ ranges from 0 to 40. The t-test (or non-parametric equivalent) will be used to determine if there is any difference in total difficulties before and after accessing the drop-in service. If providing support in this way is effective, we would expect to see a reduction in the total difficulties score (higher total difficulties score indicates a higher risk of diagnosis of mental health disorder).

All efforts will be made to ensure that the primary outcome data is collected for all patients, whether or not they complete their allocated intervention. Missing baseline data are not anticipated since baseline data are completed prior to treatment allocation. All patients with reported outcome data will be included in the analysis. If there is substantial missing data reasons for this will be explored and a sensitivity analysis will be undertaken to investigate the validity of the missing at random assumption.

### Secondary outcomes

Separate analyses will be performed for the other outcome measures (PedsQL, PHQ-9 and GAD-7), as well as the Impact subscale on the SDQ and the CSQ-8. Scores will be compared at baseline and 6-month follow up using t-tests or non-parametric equivalent analyses. Sensitivity analysis will also be conducted to look at parent-reported versus self-reported scores and their changes.

### Methods for additional analyses (e.g. subgroup analyses) (20b)

We also anticipate conducting sub-analysis on the group of participants who have received low intensity CBT to see the effect of receiving this specific intervention. Additional analysis may be conducted using demographic information collected throughout the study (such as the effect of specific LTCs, age, gender, ethnicity and parent and sibling demographics). Moderation analysis will also be conducted looking at the different sites and also considering practitioner seniority and experience.

### Interim analyses (21b)

No interim analyses are planned by the research team. Regular reports will be provided to the wider study team.

### Oversight and monitoring

Throughout the course of the project, “All Sites” meetings will be set up to check-in with the hospitals that are taking part in the study. These meetings provide an opportunity for the research team to understand the how the mental health drop-in service has been implemented at each hospital and to monitor the development and progress of the project at each site. The independent researcher will also meet with the individual sites on a regular basis to check recruitment figures, intervention allocations and to discuss any problems or queries.

### Dissemination plans

Best-practice recommendations will be used as a guide for the dissemination process. CYP, parents/carers, policy makers and PPI groups will be approached to share knowledge about the findings and importance of implementing mental health drop-in services in England. We aim to publish findings in peer-reviewed journals and engage with professionals and researchers at conferences.

## Discussion

The purpose of this protocol is to establish the objectives, methodology and overall framework for executing a study to assess the clinical effectiveness of mental health drop-in services at paediatric hospitals for CYP in the context of LTCs. Due to the current lack of mental health support for this population of patients, identifying effective services that can be implemented for this group is of high importance. Findings from this research can inform policy makers and health care professionals on how to best integrate physical and mental health support into existing services. Significant positive findings could lead to engaging the public, policy makers and clinical staff to incorporate mental health drop-in services offering low intensity CBT into routine practice within paediatric hospitals across the UK.

### Trial status

The current status of the trial is ongoing, with recruitment open from 16^th^ November 2022 – 3^rd^ January 2024 and data collection finishing in July 2024.

## Supporting information

S1 Checklist SPIRIT 2013 checklist: Recommended items to address in a clinical trial protocol and related documents*. (DOC)

S1 File (DOCX)

## Abbreviations

CYP: Children and young people
LTC: Long term condition
CBT: Cognitive behaviour therapy
CI: Chief Investigator
SDQ: Strengths and Difficulties Questionnaire
PHQ-9: Patient Health Questionnaire
PedsQL: The Pediatric Quality of Life Inventory
GAD-7: Generalized Anxiety Disorder Assessment
CSQ: Centre Satisfaction Questionnaire (CSQ)
