# Supplementary material for: Clinical effectiveness of drop-in mental health services at paediatric hospitals: A non-randomised multi-site study for children and young people and their families – study protocol
Source: PLoS One. 2024 May 9;19(5):e0302878. doi: 10.1371/journal.pone.0302878 (PMC11081357; doi:10.1371/journal.pone.0302878)
Supplement: S1 File — (DOCX) [file pone.0302878.s002.docx]

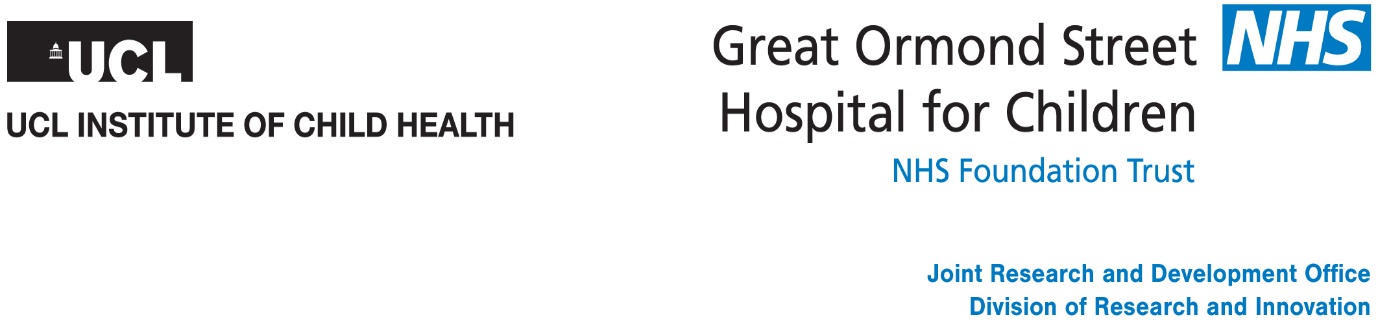
**1. Title Page**

**Study Title:** Psychological Wellbeing Drop-In Centre at Great Ormond Street Hospital for Children (GOSH): A service development research project.

**R&D Number:** 16HN11

**IRAS ID:** 213733

**Protocol Version: 5**

**Protocol Date:** 29/09/2022

**Short Title:** Psychological Wellbeing Drop-In Centre

**Chief Investigator:**

Dr Isobel Heyman

Consultant Child and Adolescent Psychiatrist

Great Ormond Street Hospital

Great Ormond Street

London

WC1N 3JH

**Investigators:**

Professor Roz Shafran

Professor of Translational Psychology and Honorary Consultant Clinical Psychologist

UCL Great Ormond Street Institute of Child Health

30 Guilford Street

London

WC1N 1EH

Dr Anna Coughtrey

Clinical Psychologist

Great Ormond Street Hospital

Great Ormond Street

London

WC1N 3JH

Dr Sophie Bennett

Clinical Psychologist

UCL Great Ormond Street Institute of Child Health

30 Guilford Street

London

WC1N 1EH

**Sponsor:** **Great Ormond Street Hospital for Children NHS Foundation Trust Joint R&D Office GOSH/ICH**, based at UCL Great Ormond Street Institute of Child Health, 30 Guilford Street, London, WC1N 1EH, United Kingdom

**Study Site:** Great Ormond Street Hospital NHS Foundation Trust

**2. Contents**

1. Title Page
2. Contents
3. Abbreviations and Definitions
4. Study Synopsis
5. Background
6. Specific Aims of the Study
7. Study Design
8. Participants
9. Recruitment and Informed Consent
10. Procedure
11. Statistical Considerations
12. Withdrawal of Participants
13. Ethical Considerations
14. Access to Personal Data
15. Participant Confidentiality and Data Protection
16. Finance and Insurance
17. Publications and Dissemination

**3. Abbreviations**

| CAMHS | Child and Adolescent Mental Health Service |
| --- | --- |
| DAWBA | Development and Wellbeing Assessment |
| GAD-7 | Generalised Anxiety Disorder Questionnaire |
| GOSH | Great Ormond Street Hospital |
| GP | General Practitioner |
| NHS | National Health Service |
| PHQ-9 | Patient Health Questionnaire |
| R&D | NHS Trust R&D Department |
| SDQ | Strengths and Difficulties Questionnaire |
| Peds-QL | Pediatric Quality of Life Inventory |
| UCL | University College London |

# Study Synopsis

| Title |  | Psychological Wellbeing Drop-In Centre at Great Ormond Street Hospital: A Service Development Research Project. |
| --- | --- | --- |
| Sponsor name |  | Great Ormond Street Hospital for Children NHS Foundation Trust |
| Primary objective |  | Do young people and their parents/carers find a drop-in centre for emotional/behavioural health at Great Ormond Street Hospital useful? |
| Secondary objective (s) |  | To evaluate a drop-in centre in terms of (i) the number of people accessing such a service, (ii) satisfaction, (iii) usefulness and, where possible, (iv) impact on physical health and (v) value for money. |
| Study Design |  | Questionnaire study |
| Study Endpoints |  | The final follow-up (6 months) of the final participant. |
| Sample Size |  | 5000 |
| Summary of eligibility criteria |  | *Inclusion criteria:* Patient at participating sites or carer or family member of a patient at participating sites.  *Exclusion criteria:* Patient must have attended one of the participating sites within the last six months. |
| Procedures: Screening & enrolment |  | The signs for the drop-in centre will make it clear that this is a research project. Young people and families may make the first approach to the research team who will sit in the centre, or the clinical research team may approach potential participants in public areas of the hospital by handing out leaflets to advertise the research. In addition, where interest is expressed and consent given, members of a patient’s direct clinical care team may sign-post or refer young people and parents/carers to the centre. No patient identifiable information will be accessed by the research team in order to approach potential participants in the hospital. Potential participants will be given written and verbal information about the project and will have the opportunity to discuss the research and ask any questions. Written informed consent (and assent where applicable) will be taken by a member of the clinical research team where appropriate. Participants may also wish to give consent via email or over the telephone if they have made the first contact with the research team by these means (e.g. after reading an advertising leaflet or poster for the study). |
| Clinical Intervention |  | The drop-in centre is funded as a research project and is currently not part of existing clinical services. If the project is successful and the centre is well-received, acceptable and effective then it will be incorporated into routine clinical practice. Therefore, we are viewing the intervention provided by the centre as research.  The intervention will vary depending on individual needs but is likely to include assessment, signposting to charities and support agencies and provision of evidence based low-intensity psychological interventions for common problems e.g. anxiety, sleep problems and behavioural difficulties.  The drop-in centre will be staffed by a qualified Psychological Wellbeing Practitioner, trained volunteers, a clinical psychologist and members of GOSH clinical teams (e.g. paediatric liaison) as appropriate. All staff will receive regular supervision from an experienced clinical psychologist, and will have access to the duty psychiatrist. |
| Measures |  | Mental health screening measures:   - Strengths and Difficulties Questionnaire (SDQ) - Patient Health Questionnaire (PHQ-9) - Generalised Anxiety Disorder Questionnaire (GAD-7) - Pediatric Quality of Life Inventory (Peds-QL)   Drop-in Centre Satisfaction Questionnaire  ONCE OFF – activity feed-back form |
| Follow up Measures |  | Mental health screening measures:   - Strengths and Difficulties Questionnaire (SDQ) - Patient Health Questionnaire (PHQ-9) - Generalised Anxiety Disorder Questionnaire (GAD-7) - Pediatric Quality of Life Inventory (Peds-QL)   Drop-in Centre Follow-Up Questionnaire (6 months after initial contact with centre). |
| End of Study |  | End of study is the last follow-up of the final participant. |

1. **Background**

Young people with physical illnesses are at significantly greater risk of developing emotional and behavioural problems than the general population. Such problems not only impact significantly on young people and their families, but can also have negative consequences for the management and course of the physical illness. Psychological treatments are highly effective for young people and adults but they are difficult to access. Parents and carers have also said that they find it difficult to access appropriate support for themselves when their child is physically unwell. There has been a recent drive to integrate physical and mental health care to improve access to evidence based psychological treatments.

The overarching aim of this research is to establish the usefulness of a Psychological Wellbeing Drop-In Centre at Great Ormond Street Hospital (GOSH). We aim to deliver a friendly, accessible drop-in centre at the heart of public areas at GOSH where young people who are patients at the hospital and their carers can access advice about common emotional, behavioural and mental health problems. Families accessing the drop-in centre may receive a range of support from a team of trained mental health professionals including:

- Evidence based assessment of mental health difficulties
- Signposting to relevant services and third sector organisations
- Information and resources about common emotional and behavioural difficulties
- ‘Low intensity’ evidence based interventions for mild mental health problems e.g. anxieties, behavioural management and sleep problems.

The drop-in centre is not currently an existing service but if this project is successful and the centre is well-received, acceptable to patients and effective then it will be incorporated into routine clinical practice.

**6. Specific Aims of the Study**

The aim of this study is to offer children and young people, and their families, quick and effective advice on their wellbeing and mental health needs. We are trying to improve the mental health and psychological wellbeing of any child attending participating sites who needs this support and ensure that any mental health needs are detected, diagnosed and treated.

The principal research question is: Do young people and their parents/carers find the drop-in centre for emotional/behavioural health at participating sites useful?

The secondary objectives are to evaluate the drop-in centre in terms of (i) the number of people accessing such a service, (ii) satisfaction, (iii) usefulness and, where possible, (iv) impact on health and (v) value for money.

1. **Study Design**

This is a feasibility study that is designed to assess user satisfaction and usefulness. All participants will receive the intervention from the drop-in centre in addition to treatment as usual.

Young people and parents/carers who approach the drop-in centre, or people in public areas of the hospital that are approached by the clinical research team and given a leaflet about the research, will be invited to participate. In addition, where interest is expressed and consent given, members of the patient’s direct clinical care team may sign-post or refer young people and parents/carers to the centre.

We will collect baseline data about young people and parent’s mental and physical health (SDQ, PHQ-9, GAD-7 and PedsQL measures) prior to their use of the drop-in centre in order to characterise the sample and identify the most common presentations.

The drop-in centre will provide evidence based information and support about common mental health difficulties. This will include assessment of needs, signposting to charities and support agencies, referral to other services and provision of low intensity psychological intervention. Low intensity means the most effective, but least intrusive, time consuming and expensive intervention. As the centre is funded as research, we are viewing these clinical interventions delivered by the drop-in centre as research.

Following their use of the centre, participants will be asked to complete brief questionnaire/s to provide feedback on the service they received (Drop-In Centre Satisfaction Questionnaire and/or the ONCE OFF – activity feed-back form). They will also be asked for permission to contact them in six months to complete additional mental health measures (SDQ, PHQ-9, GAD-7 and PedsQL) and to further establish the usefulness of the Drop-In Centre in terms of further action (e.g., did they follow up on any of the information given), impact on physical health and use of physical or mental health services (Drop-In Centre Follow Up Questionnaire).

The number of people using the pod over the period of the project will be assessed.

1. **Participants**

All young people and parents/carers that either approach the Psychological Wellbeing Drop-In Centre or are approached by the clinical research team at GOSH will be potential participants.

## Sample Size

5000. In 2014/2015, GOSH had more than 240,000 patient visits a year (outpatient appointments and inpatient admissions) and each of those patients will be accompanied by a carer bringing the total number of people eligible to access the pod to approximately half a million. If only 1% of these access the pod, this would translate to approximately 5000 per annum or 100 people per week.

## Inclusion Criteria

Current patient at participating site, or carer of a patient at participating site.

## Exclusion Criteria

Patient must have attended their participating site within the last six months. Children/families who do not speak/understand English sufficiently well to access the resources and questionnaire measures will be excluded from this study. This is primarily due to limited funding capacities for interpreters, however, should sufficient additional funding by obtained, then we would seek to recruit these families and to hire interpreters to allow equitable access to the intervention.

# Recruitment and Informed Consent

The signs for the drop-in centre will make it clear that this is a research project. Young people and their families may make the first contact with the research team, or the clinical research team may approach potential participants and give them a leaflet to inform them of the study. No patient identifiable data will be accessed by the research team in order to approach potential participants. It is possible that members of their direct clinical care team may signpost or refer them to the drop-in centre if they think that the family may benefit from taking part in the research. Any young person or parent/carer that enters the drop-in centre will be invited to participate by a member of the clinical research team. They will inform the family about the study and give written information to interested participants. As there are no strict inclusion/exclusion criteria no medical records will be screened by the research team at this stage.

The appropriate information sheet will be given to all potential participants, informing them about the study, and what participation will involve. An information sheet will be given both to the young person and their parent/caregiver if they are under 16. Separate REC approved information sheets will be provided for parents/carers and young people of different age ranges. They will be provided with contact details of the research team should they wish to discuss this further or ask any questions. They can choose to consider participation and return another time and there will be no expectation that potential participants will consent on their first arrival at the centre.

Participants will be given the opportunity to ask any questions and only once all of their queries have been answered will consent be taken by the principal investigator or suitably qualified, delegated member of the clinical care team.

We will obtain informed consent from all participants prior to taking part in the study. Written informed consent will be received from most participants. However, some participants may wish to consent via email or telephone if they have made first contact with the research team by these means (e.g. after picking up an advertising leaflet). Participants will then be invited to attend the drop-in clinic where they will follow the usual procedure including assessment, completion of questionnaire measures and provision of low-intensity interventions. Some of these procedures may be completed remotely, as per usual procedures (e.g. one possible intervention may be guided self-help provided over the telephone).

If the participant is not able to consent for themselves, they will provide informed written assent and a parent/guardian will provide written informed consent on their behalf.

We will make it clear to all potential participants that their usual care will not be affected by their decision to participate. A list of services and information about what to do in an emergency (e.g. access to out-of-hours GP and A&E) will be available to all potential participants.

**10. Procedure**

Mental Health Measures

Following written consent, participants will be asked to complete up to three brief screening measures of mental health as follows:

**SDQ:** This is a brief screening questionnaire for common mental health problems in young people. It will be completed by parents only if the child is under 11 years, or by both the parent/carer and child if the child is aged 11 years or over. It will be completed in the drop-in centre

**PedsQL**: This is a brief assessment measure of young people’s quality of life. It will be completed by the parent/carer only if the child is under 8 years, or by both the parent/carer and child if the child is aged 8 years or over.

**PHQ-9:** This is a brief screening measure for depression in adults. It will be completed by parents/carers to assess parental mental health for children under 12. Children 12 years or over will complete this measure to assess child mental health.

**GAD-7:** This is a brief screening measure for anxiety in adults. It will be completed by parents/carers to assess parental mental health for children under 12. Children 12 years or over will complete this measure to assess child mental health..

The SDQ, PedsQL, PHQ-9 and GAD-7 are all routinely used in clinical practice.

Participants will complete these measures either in the clinic, over the telephone, via email or online in Redcap according to patient preference.

Planned Interventions

Families will have the opportunity to outline their concerns to a trained mental health professional. Depending on the nature of the difficulty, possible outcomes might be:

- Standardised assessment using web-based, evidence-based brief measures including opportunity to access a widely used standardised online detailed assessment of child mental health (Development and Wellbeing Assessment; DAWBA) and obtain an assessment report;
- Provision of information or self-help material/website suggestions;
- Signposting e.g. where to find appropriate support agencies, charities etc.;
- Referral to appropriate services either within Paediatric Psychology or Psychological Medicine at participating sites, Psychiatry at participating sties or in the local vicinity of the family; and/or
- Brief focussed evidence-based advice for common mental health problems e.g. behaviour management, childhood fears, sleep problems.

Care Pathways

The drop-in centre will be staffed by (1) a member of the clinical research team qualified to deliver low intensity mental health interventions (a qualified ‘psychological wellbeing practitioner’); (2) a member of participating site or volunteer team who will have undergone prior training; and (3) depending on need and demand, members of the psychology, psychiatry, mental health and psychological teams at participating site who will provide sessional input to the centre.

All members of staff will have regular supervision by a qualified clinical psychologist. The duty psychiatrist for the hospital will be available at all times. The service would be closely linked to psychiatric liaison, paediatric psychology and the CAMHS department.

The drop-in centre will offer a ‘first step’ on the stepped care pathway. Some children and/or carers may be identified as having a significant mental health disorder warranting urgent/immediate treatment. Children/carers identified as having psychiatric symptoms will be referred to services as appropriate. Thus, children/carers who screen for a severe mental health disorder or whose screening questionnaire is otherwise suggestive of risk will be referred to other services as appropriate, consistent with best practice guidance. The research team comprises a number of trained clinicians, who will be well placed to liaise with, and refer to, other services. This may include (although is not restricted to) local CAMHS, the participating site. Psychological Medicine Team or Paediatric Psychology. This procedure will also be followed should it become apparent that there is risk (including harm to self and others) during the course of the research. Liaison will continue until the risk is adequately managed.

Satisfaction and Follow-Up

Following use of the Drop-In Centre, participants will be asked to complete the Drop-In Centre Satisfaction Questionnaire and where a single-session intervention was provided: the ONCE OFF – activity feed-back form. These will be completed by the parent/carer only if the child is under 11 years, or by parent/carer and child if child is aged 11 years or over (8 years or over for the ONCE OFF – activity feed-back form). Participants may also be interviewed about their experience of trying to access support, their pathway to the centre and their experience of the support provided.

We will ask permission from all participants to contact them again in six months time to ask them for additional feedback on the centre. The Drop-In Centre Follow-Up Questionnaire will be sent by post or email according to participant preference six months after their first contact with the Centre. This will be completed by the parent/carer only if the child is under 11 years, or by parent/carer and child if child is over 11 years. It will be completed in participants own home and returned by post or email according to participant preference.

Medical records will be reviewed to characterise the nature of the intervention received from the drop-in centre, and number of appointments or referrals to other services during the six month follow-up period. Written consent will be sought prior to anyone outside of the direct clinical care team gaining access to participants' personal data.

## 10.1. Participant reimbursement

Participants will not receive any payments, reimbursement of expense or any other benefits or incentives for taking part in this research.

**10.2. Study Duration**

The study will last for 2 year, 11 months and 30 days in total (02/01/2017-31/12/2019). The study duration for each participant will be a maximum of 6 months (from initial drop-in to following up on impact of drop-in centre 6 months later).

**10.3. Definition of End of Study**

The end of study is the date of the last contact (i.e. 6 month follow-up) for the last participant.

1. **Statistical Considerations**

The number of people using the service and their feedback on it are the primary outcome measures for the feasibility study. This will include mean satisfaction ratings and written descriptions of the usefulness of the drop-in centre.

We will also look at the mean scores on the SDQ, PedsQL, PHQ-9 and GAD-7 in order to characterise the sample.

We will review medical records (with participants permission) over a six month period to categorise (a) the nature of the contact with the drop-in centre; (b) number of referrals to other services; and (c) number of hospital appointments over the six month follow-up period.

We will use t-tests and repeated measures analysis of variance to compare changes in mental and physical health over the six month follow-up period.

## 12. Withdrawal of Participants

Discontinuation criteria: The research will be stopped prematurely if a significant proportion of participants do not consent to participate.

Participants will be free to withdraw from the study at any time, without impact on their clinical care.

1. **Ethical Considerations**

Taking part in the research will be in addition to usual care. No participant will be deprived of usual treatment. Information about who to contact in an emergency, information about how to recognise signs of risk and a list of available services will be available to all families regardless of whether they decide to participate in the study.

Some children and/or carers may be identified as having a significant mental health disorder warranting urgent/immediate treatment. Children/carers identified as having psychiatric symptoms will be referred to services as appropriate. Thus, children/carers who screen for a severe mental health disorder or whose screening questionnaire is otherwise suggestive of risk will be referred to other services as appropriate, consistent with best practice guidance. The research team comprises a number of trained clinicians, who will be well placed to liaise with, and refer to, other services. This may include (although is not restricted to) local CAMHS, the participating site’sTeams or Paediatric Psychology. This procedure will also be followed should it become apparent that there is risk (including harm to self and others) during the course of the research. Liaison will continue until the risk is adequately managed.

All children, young people and families will be given details of who to contact in an emergency and information about how to recognise signs of risk. Participants will be fast-tracked to appropriate services should significant risk to self or others (including safeguarding concerns) be identified, in accordance with best practice guidance.

It is possible that talking about mental health needs and completing the questionnaires may cause participants/carers to become distressed. The research team is made up of a number of clinically trained researchers, who will be well placed to manage this distress should it arise, and to signpost/refer to sources of support (such as local mental health services) if necessary.

**14. Access to Personal Data**

During the study, the participants’ direct care team (including involved health professionals within participating site; GP; other involved health and education professionals where appropriate) and the research team will have access to the participants’ personal data.

Details of the clinical intervention provided will be recorded on patient medical records. Members of the research team may also need to access medical records in the unlikely event that a risk or safeguarding issue arises during the course of the research. They will also access the medical records from the drop-in centre to characterise the nature of the intervention received and any outcomes (e.g. referral to other service), including number of hospital appointments during the 6 month follow up period. Written consent will be taken from participants to access medical records.

Postal and email addresses will be required to write to participants to send them follow-up questionnaires. Phone numbers will be required in the event that a significant mental health need is identified or if a participant is distressed to provide further resources or possibly a low intensity telephone intervention. The research team will only have access to these details if the participant has consented to this.

**15. Patient Confidentiality and Data Protection**

Personal data will be contained within a trial master file (paper copy), which will be kept in a locked cabinet accessible only to the research team. All electronic information will be kept in encrypted, password protected files on NHS or university computers and the UCL Information Data Safe Haven. Identifiable information, such as names and addresses will only be stored on password protected NHS computers. Personal data contained on research databases (for example participant contact addresses) will be kept in a separate database to study data (e.g. outcome measures). All electronic information will be kept in encrypted, password protected files stored on the UCL Information Data Safe Haven.

Some participants may choose to complete their study outcomes online on the Redcap database. REDCap, developed by Vanderbilt University, is an electronic data capture and transfer software. The software is hosted on virtual servers provided by AIMES (an ISO27001 certified cloud hosting provider) in its own data centre in Liverpool, UK. The software is licenced for use by Vanderbilt University to REDCap consortium members, of which GOSH is one. The study data will be stored on secured virtual Linux servers (specifically, GOSH-REDCap-DB01, GOSH_REDCap-WB01) with an appropriate level of encryption based in England, UK.

Each participant will be allocated a unique study number at study entry and will be identified by this number on all study related documentation throughout the course of the intervention and data analysis process. Any data transferred will be done according to the NHS Code of Practice on Confidentiality.

**15.1. Data Storage**

Personal data will be stored for less than three months after the study has ended. Research data generated by the study will be stored for 15 years.

**15.2. Archiving**

All study related documentation and data will be archived in accordance with the Sponsor’s SOPs, Policies and Procedures.

**16. Financial Information and Insurance**

Cover for negligent harm will be provided by the Great Ormond Street Hospital for Children NHS Foundation Trust through the Clinical Negligent Scheme for Trusts (CNST). No-fault compensation insurance cover for any non-negligent harm will be provided by University College London.

# 17. Publications and Dissemination

The results of the study will be reported and disseminated as follows;

- Peer reviewed scientific journals;
- Internal report;
- Conference presentation(s);
- Publication on website; and
- Internal newsletters.
